# Cyclic Lipopeptides from *Bacillus subtilis* ABS–S14 Elicit Defense-Related Gene Expression in Citrus Fruit

**DOI:** 10.1371/journal.pone.0109386

**Published:** 2014-10-15

**Authors:** Waewruedee Waewthongrak, Wichitra Leelasuphakul, Greg McCollum

**Affiliations:** 1 Department of Biochemistry, Faculty of Science, Prince of Songkla University, Hat Yai, Songkhla, Thailand; 2 United States Department of Agriculture-The Agricultural Research Service, U.S. Horticultural Research Laboratory, Fort Pierce, Florida, United States of America; Università della Calabria, Italy

## Abstract

Effects of cyclic lipopeptides (CLPs) obtained from *Bacillus subtilis* ABS-S14 on eliciting defense-related gene transcription and activity of defense-related enzymes; glucanase (GLU), chitinase (CHI), peroxidase (POX) and lipoxygenase (LOX) in *Citrus sinensis* cv. Valencia fruit were determined. The maximum level of *GLU* transcripts induced in fruit treated with fengycin was significantly greatest among treatments at 48 h. Surfactin enhanced the *LOX* and *POX* transcripts. In parallel, corresponding enzyme activities were correlated with changes in gene expression observed in fruit inoculated with *Penicillium digitatum* following treatment with individual CLPs. Synergistic effects of fengycin and iturin A, fengycin and surfactin were shown in gene transcript of *GLU* and *CHI*, respectively, and surfactin induced *POX* and *LOX* gene expression of citrus flavedo without pathogen infection. These results suggest that fengycin and surfactin act as elicitors of defense-related gene expression in “Valencia” fruit following infection.

## Introduction

Many *Bacillus* species are known to be antagonistic microorganism because of their potential to produce several structurally diverse antimicrobial compounds that act against the growth of plant pathogens [1]. Among the broad spectrum of biologically active molecules synthesized by *Bacillus*, some of them have antagonistic activity against plant pathogens. This antagonistic activity results from the production of antibiotics and has been well documented as being an important mechanism that can limit the growth of pathogen within host plant tissues [2]. Recently, cyclic lipopeptides (CLPs) have been identified as a major group of compounds produced by *Bacillus* spp. that are antagonistic against the growth of pathogens. There are three main families of CLPs: iturin A, fengycins, and surfactins, each of which may display strong antimicrobial activity, but against different pathogens [3]. Their structures consist of a variable hydrophobic alkyl chain and peptide ring of unusual amino acids including D-, O-methyl, N-methyl and β-amino acids [4]. Each CLP family also consists of variants each with the same peptide length, but with different amino acid residues at specific positions. These peptide variants showed different biological activities. Strains of *Bacillus subtilis* that produce iturin A and fengycin have been shown recently to control brown rot caused by *Monilini*a spp. in peach [5]. Fengycins have antagonistic activity against a broad range of fungal targets and have also been shown to elicit induced systemic resistance (ISR) in plant hosts. Surfactins are also elicitors of ISR in plants [6]. Surfactins and fengycins purified from *Bacillus* have been shown to induce systemic resistance [7].

Production of pathogenesis-related proteins (PRs) is among the initial responses of plants to infection by pathogenic fungi. PRs have been shown to protect plants from pathogen attack. A large number of PRs associated with ISR, are enzymes such as peroxidase (POX), lipoxygenase (LOX), β-1,3-glucanase (GLU), and chitinase (CHI). LOXs catalyze dioxygenation of linoleic acid (18∶2) and linolenic acid (18∶3) into hydroperoxide compounds which can be metabolized to jasmonic acid (JA), and play important roles in plant defense [8]. POXs are well known enzymes in protecting cells from damage by changing free radicals and reactive oxygen species to phenolic compound, H_2_O and O_2_. Consequently, these compounds have been shown to be involved in various physiological processes, such as lignification, suberization, auxin catabolism, wound healing and defense mechanisms against pathogen infections [9]. The role of POXs and LOX associated with ISR pathway which regulated by jasmonate and ethylene has been widely demonstrated [10]. Chitinases and β-1,3-glucanases are hydrolases which are often found in higher plants, and they provide a general defense response after wounding/or pathogenic attack [11]. Results obtained from numerous studies have demonstrated that chitinase and β-1,3-glucanase probably protect plants from pathogenic fungus attack by degradation of chitin and β-1,3-glucans which are important structural components of many fungal cell walls. Efficacy of *Bacillus* CLPs in inducing a set of PR genes and established ISR has been demonstrated [12].

Recently, it was reported that crude extracts prepared from antagonistic strains of *B. subtilis* exhibited strong antifungal activity and gave reasonable protection of citrus fruit from green mold decay caused by *Penicillium digitatum* compared to the chemical fungicide [13]. Green mold is perhaps the most important postharvest pathogen of citrus fruit and can lead to significant economic losses. Typically, citrus fruit are treated with fungicides following harvest to reduce the incidence of green mold as well as other postharvest decay problems. However, resistance to fungicides, as well as public health and environmental concerns, necessitate the development of alternative treatments to control postharvest decay. Some success at reducing green mold decay of citrus has been achieved with biological control agents. The effectiveness of an antagonistic *B. subtilis* ABS-S14 alone or in combination use with yeast (*Pichia guilliermondii*) to control green mold disease cause by *P. digitatum* in citrus was recently demonstrated [14]. Moreover, fengycin and surfactin are the major CLP antibiotics present in crude extracts of *B. subtilis* ABS-S14 culture medium were responsible for the inhibitory activity of *B. subtilis* against *P. digitatum*. After treatment mandarin fruit with the antagonistic bacteria, its crude antibiotics and chitosan the reduction of *P. digitatum* induced fruit decay was detected [15]. However, the mechanism of this effect has not been accomplished yet. The aim of this study was to determine the effects of CLPs in crude extracts of *B. subtilis* ABS-S14 on elicitation of pathogenesis related protein transcripts and related enzyme activities in citrus flavedo. Abundance of gene transcripts and enzyme activity of POX, CHI, GLU and LOX were measured. Roles of individual CLPs in activation of these defense related proteins in the citrus tissues with and without pathogen infection, including their synergistic effects were also investigated.

## Materials and Methods

### Microorganisms

In this experiment the field studies did not involve any endangered or protected species. Antagonistic *B. subtilis* ABS-S14 was isolated from soil collected from citrus groves around the south of Thailand where there is no specific permissions were required for these locations/activities. The lands are private properties located as follow:

Khiriwong, Phrom Khiri District, Nakhon Si Thammarat 80320, Thailand

Latitude: 8.52144 | Longitude: 99.82514; Altitude: 30 meters.

All the single colonies of *B. subtilis* ABS-S14 were cultured on nutrient agar (NA) plates at 37°C for 18 h. *B. subtilis* ABS-S14 was characterized by Gram staining, cell shape with the presence of spores and bio-chemical identification [14].

*P. digitatum* was isolated from decayed citrus fruit, and was maintained on PDA plates, and kept at 25°C. The fungus was routinely transferred to surface-sterilized fruit for maintaining pathogenicity. *P. digitatum* conidial suspensions were prepared from a 7 day-old culture. The concentration was determined with a haemocytometer using a compound light microscope (40×), and its concentration was adjusted to 10^4^ spores/mL by dilution with sterile distilled water.

### Extraction of the *B. subtilis* crude extract and purification of CLPs

A crude extract was prepared from a 3 day-old *B. subtilis* cell free supernatant by ethanol extraction. Liquid cultures were prepared with shaking (200 rpm) at 37°C for 72 h, cell free supernatant was collected by centrifugation at 10,000×g for 15 min, supernatants were decanted and the pH was adjusted to 2.0 with 6 N HCl. After centrifugation at 15,000×g, 4°C for 20 min, the precipitate was extracted twice with 80% ethanol and taken to dryness by rotary evaporation at 65°C. The dried residue was weighed, and subsequently re-dissolved in 80% ethanol (crude extract). CLPs were purified from the crude extract using preparative thin-layer chromatography (PTLC) on Silica gel 60 F_254_ 1 mm thicknesses, 20×20 cm, (Merck, Germany). A PTLC mobile phase consisted of chloroform-methanol-water (65∶25∶4 v/v). CLP bands were detected under UV light and subsequently sprayed with water. The CLP bands were recovered from silica gel by ethanol extraction and the antifungal activity of the CLPs was tested using disc diffusion assay [13].

### Reverse Phase-High Pressure Liquid Chromatography (RP-HPLC) analysis

Identification of *B. subtilis* ABS-S14 CLPs was performed by the HPLC (Agilent 1200, Agilent Technologies Inc., USA) which was equipped with a LiChrospher 100 RP-18e column (4.6×100 mm, 5 µm, Merck, Germany). The mobile phase consisted of a mixture of 0.1% (v/v) trifluoroacetic acid in MiliQ water and acetonitrile. A stepwise gradient elution was conducted as follows: 35% acetonitrile at 0 min to 80% acetonitrile at 60 min. The flow rate was set at 1.0 mL/min and absorbance was monitored at 210 nm with photodiode array detector.

### Matrix-Assisted Laser Desorption Ionization Time-Of-Flight Mass Spectrometry (MALDI-TOF MS) analysis

To characterize and identify CLPs produced by *B. subtilis* ABS-S14, MALDI-TOF MS was used and recorded using a Bruker Daltonic Ultraflex III MALDI-TOF/TOF instrument with a 337 nm nitrogen laser for desorption and ionization at the Bioservice Unit, National Center for Genetic Engineering and Biotechnology (BIOTEC) under National Science and Technology Development Agency (NSTDA), Thailand. The matrix was α-cyano-4-hydroxycinnamic acid. The lipopeptides were detected in the range of molecular ion peaks from m/z 900 to 1800.

### Plant material

*Citrus sinensis* “Valencia” fruit of uniform size and maturity were harvested from a commercial orchard in St. Lucie County, Florida, USA with no specific permissions were required for these locations/activities. The location is specified as follow: private citrus grove at Latitude: 27.44459, Longitude: −80.338962, Altitude: 5 meters. Fruit were immersed in 1% sodium hypochlorite, and then rinsed with tap water and air dried at room temperature. Five wounds in a circle of 0.5 cm diameter and 3 mm depth were made by puncturing the fruit rind on the equator of the fruit with a sterile needle.

### Effects of CLPs on induction of defense related gene transcript and enzyme activity

Two experiments were conducted to determine the effects of CLPs on defense-related gene transcript abundances and related enzyme activities. The first experiment was conducted to determine the effects of CLPs in citrus fruit that were challenged with *P. digitatum*. Five treatment groups were included in this experiment: fruit treated with (1) a conidial suspension of *P. digitatum* (Pd), (2) iturin A and Pd (I+Pd), (3) fengycin and Pd, (F+Pd), (4) surfactin and Pd (S+Pd), and (5) sterilized water as the control treatment (C). A 20 µL aliquot of each CLP (1 mg/mL) was pipetted into each wound and allowed to dry for 2 h prior to adding 20 µL of the *P. digitatum* conidial suspension.

The second experiment was conducted to determine the synergistic effects of CLPs on defense-related gene transcript abundance and enzyme activity. In this experiment, 1 mg/mL each of CLP and crude extract solution were used, fruit treatments were divided into 5 groups including; (1) fruit treated with crude extract (CE), (2) S+I, (3) S+F, (4) F+I, and (5) (C). The 20 µL aliquot of each solution was pipetted into each wound.

Following treatment fruit were sealed in a plastic box containing a cup of water to maintain a high humidity and all boxes were incubated at 25°C. At 24, 48 and 72 h following treatment, the flavedo tissues with circle 1.0 cm away from the inoculation site were taken. Tissue was frozen immediately in liquid nitrogen and stored at −80°C until the time of RNA and protein extractions.

Total RNA was extracted from 1 g of citrus flavedo by the Guanidine thiocyanate procedure [16] and was treated with DNase I (Amersham, England). Concentration and purity of RNA were determined by measuring absorbance at 260/280 nm (NanoDrop, Thermo Scientific, USA).

One g of flavedo was ground to a fine powder under liquid nitrogen. For protein extraction, 50 mg of powdered flavedo were homogenized in 1.5 mL of phosphate buffer (100 mM, pH 6.0) and subsequently centrifuged at 14,000×g at 4°C for 15 min to eliminate insoluble material. The supernatant was decanted and stored at −20°C until used for assay of enzyme activities. Total protein concentration in supernatant was determined according to the Bradford method [17] using bovine serum albumin as a standard.

### Quantitative real-time (qRT) PCR assay

Total RNA was used as template for cDNAs synthesis using the Superscript First-Strand Synthesis Kit according to the manufacturer's instructions (SuperscriptII, Invitrogen, USA). Following cDNA synthesis RNA was removed from the cDNA products by treatment with RNase H (Invitrogen, USA).

Primers for target genes; elongation factor 1 alpha (*EF-1α*), glucanase (*GLU*), chitinase (*CHI*), peroxidase (*POX*), and lipoxygenase (*LOX*) were designed using the primerQuest program based on sequence data for citrus available from GenBank. Primer sequences as presented in Table 1. The target gene sequences were amplified from 1 µL of cDNA (50 ng/µL) using the PCR master mix kit according to the manufacturer's instructions (Platinum PCR SuperMix, Invitrogen, USA). The PCR was performed under following conditions: 10 min at 95°C, and 40 cycles of 15 sec at 95°C, 30 sec at 50–60°C and 45 sec for 72°C. PCR products were analyzed on 1.5% agarose gel electrophoresis.

**Table 1:** Gene-specific primer sequences and amplified products of defense-related and reference genes.

Name	Forward (5′→3′) sequences	Reverse (5′→3′) sequences	Amplicon length (bp)
***EF-1α***	ACATGATTACCGGTGCCTCACA	ACACCAAGGGTGAAAGCAAGCA	133
***GLU***	ACCTCCGAAGAATCGCTTCCAA	TGTTTCTCATGGCGGGAACA	160
***CHI***	AATGATGAACGATGCCCTGCCA	CCACTTGATGCTGTCTCCAA	151
***POX***	AGCCAGGAGACAATGAACAG	TAGTTTCATGGCCAGTTTGGGC	188
***LOX***	GTCGTTCTGGAACTTGTCGGCACT	CTGTGATTGCACCAGGCGTCCC	179

PCR-amplified sequences were inserted into the 2.1 TOPO vector as recommended by the TOPO TA Cloning Kit manufacturer (Invitrogen, USA), and was transferred to competent *E. coli* DH5α-T1R (Invitrogen, USA) using the chemical transformation technique. Transformed bacterial cells were cultured in 250 µL of S.O.C. medium (2% tryptone, 0.5% yeast extract, 10 mM NaCl, 2.5 mM KCl, 10 mM MgCl_2_, 10 mM MgSO_4_, and 20 mM glucose), and incubated for 1 h at 37°C. For cell selection, 100 µL of the cell cultures was spread onto LB agar plates containing ampicillin (50 µg/mL) which was spread with 40 µL of X-gal (40 mg/mL), and incubated at 37°C for 18 h. Several white colonies were picked and transferred to LB broth containing ampicillin, and cultured overnight at 37°C. Plasmids were harvested using the Plasmid Miniprep Kit (MACHEREY-NAGEL Tech, USA). Plasmid concentration and purity were determined by measuring absorbance at 260/280 nm (NanoDrop, manufacturer).

Standard curves for each target sequence were developed by 10×-fold serial dilutions of plasmid. The PCR mixture was prepared from 1 µL of each dilution, 12.5 µL of 2× QuantiTect SYBR Green PCR Master Mix (Qiagen, USA), and 100 nM of each gene-specific primer in a final volume of 25 µL. PCRs with no template controls were also performed for each primer pair. The qRT-PCR was performed employing Applied Biosystems 7300 Real-Time PCR System (Applied Biosystems, USA). The PCR cycling program was started 2 min at 50°C, 10 min at 95°C, for activating the Taq DNA Polymerase, and a three-step amplification including a denaturation step at 95°C for 15 sec, the annealing step at 50–60°C for 45 sec, and the extension step at 72°C for 30 sec for a total of 40 cycles. The standard curves for each plasmid concentration were expressed as a C_T_ value (axial Y) and plotted against the logarithm of the plasmid copy number (axial X). Linear regression analysis was conducted using the Microsoft Excel program. The PCR efficiency for each primer pair was calculated by the equation E% = (10^1/slope−1^)×100 [18]. The slope was obtained from linear regression of standard curve. The percent amplification efficiency was between 90–105% for an optimized PCR reaction. Abundance of *EF-1α* transcripts in citrus was measured in various tissues of citrus, leave, root, and flower and fruit flavedo using qRT-PCR as previously described.

### Abundance of defense related gene transcripts

The transcript level was calculated from the plasmid standard curves and normalized against the *EF-1α* transcripts. For each gene, the lowest sample value in the sterile water control treatment was defined as the 1× expression level, and results were expressed as fold increases of the mRNA over the control sample [19].

### Enzyme activity assays

Chitinase activity was determined using carboxymethyl-chitin-remazol brilliant violet (Loewe Biochemica, Germany) as substrate. To conduct the chitinase activity assay, 100 µL of substrate (2 mg/mL) was mixed with an equal volume of 1 M sodium acetate buffer (pH 5.0), and incubated at 35°C for 5 min. Then, 200 µL of plant extract was added to the substrate and further incubated at the same temperature for 1 h. The enzyme reaction was stopped by adding 200 µL of 1N HCl, and chilled on ice for 10 min. Non-degraded substrate was precipitated by centrifugation at 10000×g for 10 min. The absorbance of supernatant was measured at 550 nm. Activities are reported as A550/min/mg protein (mgP).

β-1,3-glucanase activity was determined using carboxymethyl-curdlan-remazol brilliant blue (Loewe Biochemica, Germany) as substrate. To conduct the glucanase assay, 100 µL of substrate (4 mg/mL) was mixed with an equal volume of 1M sodium acetate buffer (pH 5.0), and incubated at 35°C for 10 min. Aliquot of 200 µL of plant extract was added to the substrate, and further incubated for 1 h. Enzyme activity was stopped by adding 200 µL of 1N HCl, followed by chilling on ice for 10 min. Reaction mixtures were centrifuged at 10,000×g for 10 min to discard non-degraded substrate. Absorbance of supernatants at 600 nm was measured. Activities were reported as units [A_600_/min)×1000]/mgP) [20].

Peroxidase activity was assayed by mixing 200 µL of plant extract with 1,774 µL of potassium phosphate buffer (100 mM, pH 6.0), 6 µL of 20 mM guaiacol (Merck, Germany) and 20 µL of H_2_O_2_ (30% w/w). Identical reaction mixtures without H_2_O_2_ served as a minus substrate blank. Following the addition of H_2_O_2_, absorbance at 470 nm was recorded every 15 sec for 2 min. The maximum slope of the linear phase of reaction progress curve (ΔA470/ΔTime) was used to calculate the enzyme activity and expressed as units per mg of protein (Units/mgP). One unit of peroxidase activity represents the amount of enzyme catalyzing the oxidation of 1 µmol of guaiacol in 1 min at pH 6.0.

Lipoxygenase activity was determined following the method of Chen and Whitaker [21]. Sodium linolate (10 mM) substrate was prepared by mixing 157.2 µL of linolenic acid (Sigma-Aldrich, USA), 157.2 µL of tween 20, 10 mL of distilled water, and then clarifying by the addition of 1 mL of 1N NaOH. Then, the substrate solution was made up to final volume (200 mL) by adding phosphate buffer (200 mM, pH 7.0). To conduct the LOX assay, 0.5 mL of plant extract was added to 1.5 mL of substrate. Absorbance at 234 nm was recorded every 15 sec for 5 min. LOX activity unit was expressed as ΔA234/min/mgP.

## Results

### CLP purification and their antifungal activity

**Figure 1:**
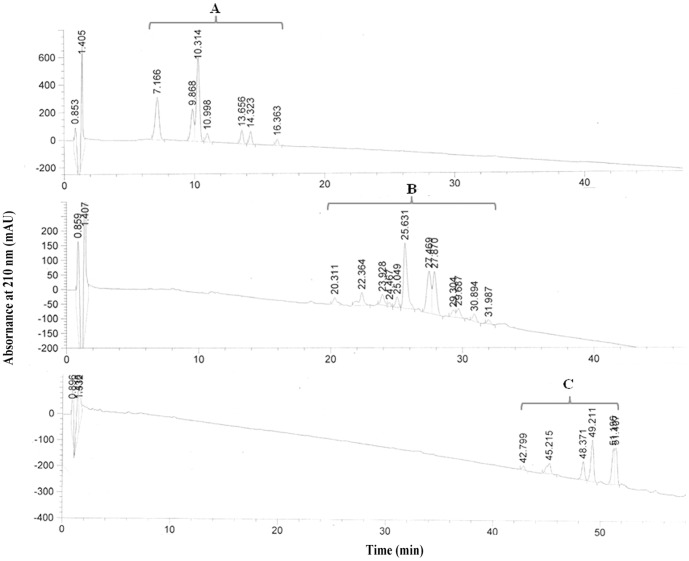
RP-HPLC profiles of partially purified CLPs from *B. subtilis* ABS-S14. Three clusters A, B and C were recorded at 210 nm.

**Figure 2:**
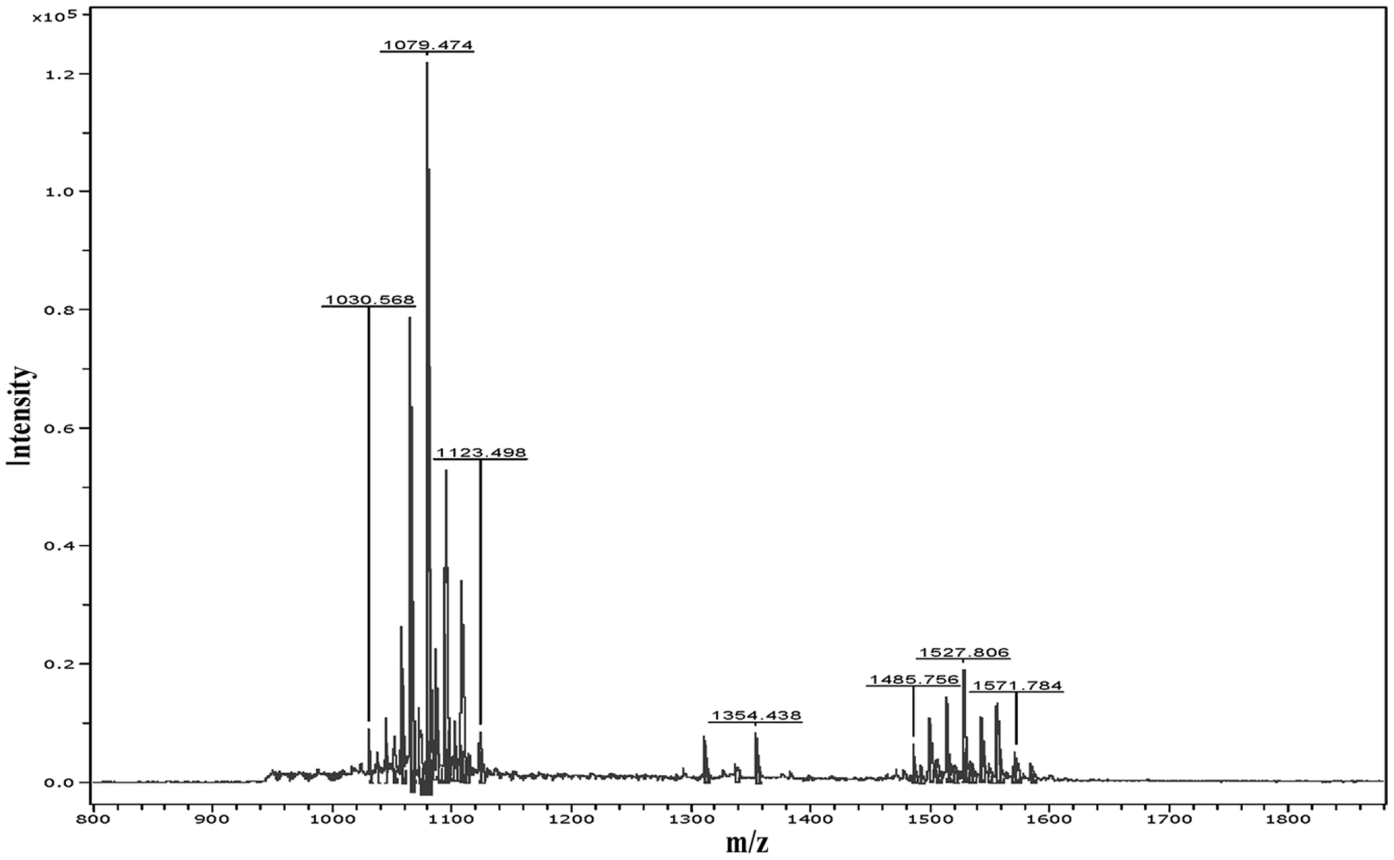
MALDI-TOF MS analysis of a crude extract produced by *B. subtilis* ABS-S14.

Three groups of CLPs produced by *B. subtilis* ABS-S14 were preliminarily detected on PTLC plates with the R_f_ values of 0.10, 0.32 and 0.62, which were corresponded to those of fengycin, iturin A, and surfactin standards, respectively. The separation and identification of CLP antibiotics was also accomplished by RP-HPLC (Figure 1). It indicated that CLPs of *B. subtilis* ABS-S14 were composed of three clusters of absorption peaks. The retention times of cluster A and C eluted at 7–16 min and 42–52 min were identical to those of the commercial (Sigma-Aldrich, USA) surfactin and iturin standard RP-HPLC chromatograms, respectively. Characterization of fengycins which was recovered in cluster B at 20–32 min retention time was based on the additional MALDI-TOF MS data of a crude extract of *B. subtilis* ABS-S14 as presented in Figure 2. It revealed the two molecular ion peaks at *m/z* 1030–1120 and 1480–1580. The coproduct ions at *m/z* 1030–1120 were attributed to the isoforms of surfactins and iturins. Ion clusters around *m/z* 1480–1580 indicated the existence of fengycin isoforms by comparing to the data illustrated in [22]. Thus, the result obtained from HPLC and MALDI-TOF MS analysis confirms that antimicrobial compounds produced by *B. subtilis* ABS-S14 consist of the well-known LPs families like iturin, fengycin, and surfactin.

Effects of the purified CLPs on growth of *P. digitatum* were determined on agar plates. The iturin A and fengycin preparations showed the most potent inhibitory effect on the growth of *P. digitatum* by 94.8 and 70.2% fungal inhibition, respectively. In contrast, no inhibition was detected in plates treated with surfactin. The green mold rot symptom was clearly observed in fruit treated with surfactin followed by pathogen inoculation, and pathogen alone on day 3. However, the average lesion diameter measured from surfactin treated wound was less than those treated with *P. digitatum* only.

### Abundance of Elongation Factor 1 alpha transcript in various tissues of citrus

It was demonstrated that the abundance of *EF-1α* transcripts is consistent in various tissues of citrus cultivars used in the experiments. The abundances of *EF-1a* transcripts were detected in citrus cultivars in the flowers, leaves, roots and flavedo from both immature and mature fruit. The C_T_ values of *EF-1a* in all the tested samples ranged from 19.8–21.1. This clearly indicated that the *EF-1a* gene expression was relatively stable in different tissues. Thus, *EF-1a* was considered to be a suitable reference gene for this work, since its expression was seemingly independent of the development stage, cell/tissue types, treatments and environmental conditions as had also been described by Øvergård et al. [23].

### Induction by CLPs on the defense related gene transcript in *P. digitatum* infected flavedo

**Figure 3:**
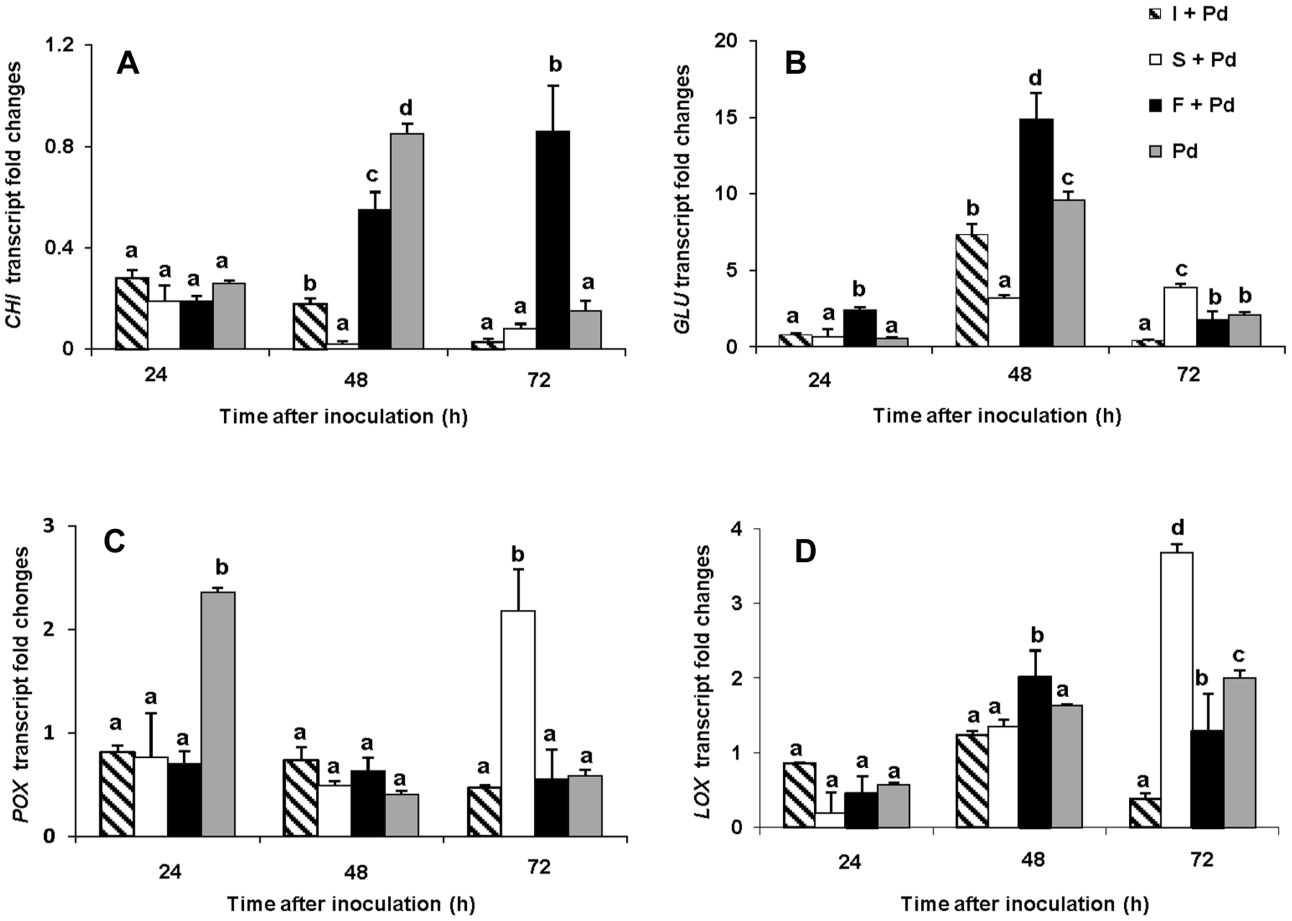
Abundance of transcripts for defense related genes induced by various CLPs in infected flavedo. Expression level of A: *GLU*, B: *CHI*, C: *POX* and D: *LOX* after wounding and co-inoculation with CLPs and *P. digitatum*. All treatments of iturin A (I), fengycin (F), and surfactin (S) had been inoculated with *P. digitatum* (Pd) (10^4^spores/mL). In each treatment, vertical bars represent standard error from mean of three replications. Columns with the same letter above them do not differ significantly (*p*≤0.05) by Duncan's multiple range test.

Abundances of *GLU*, *CHI*, *POX* and *LOX* transcripts in flavedo were quantified at 24, 48, and 72 h after treatment with *B. subtilis* CLPs and *P. digitatum*. The patterns of change in transcript abundance differed among the four genes investigated were presented in Figure 3. Abundance of *CHI* transcripts was very low with the highest level being at 48 h with infection alone (Figure 3A). Fengycin had only a minor effect on the abundance of *CHI* gene transcripts. However, in the presence of fengycin it was maintained at (0.86 fold) while it reduced to about 0.1 fold in its absence at 72 h treatment. Transcripts for *GLU* showed the greatest change in abundance amongst the genes investigated. In all treatments of CLPs co-application with the pathogen, with the exception of surfactin, high abundances of *GLU* transcripts were significantly detected, particularly in the citrus fruit treated with fengycin (Figure 3B). The increase in the *GLU* transcripts started 24 h and reached the highest expression level (15×) at 48 h after inoculation with *P. digitatum*. This maximum level of *GLU* transcript level in fruit treated with fengycin was about 1.5× fold greater than those presented in the *P. digitatum* citrus fruit. The pathogen itself elicited a similar *GLU* induction profile in the infected fruit with a maximum at 48 h (9 folds), and then was less after 72 h. In addition, the transcript level of *GLU* in the fruit treated with iturin A, co-applied with pathogen, reached a maximum at 48 h (7.5 fold), and was then significantly less at 72 h.

Among the different CLP treatments, surfactin induced the highest expression of the *POX* and *LOX* genes, with a lower effect by fengycin and iturin A. The *POX* gene became fully expressed (2.3 fold) at 72 h after the fruit were treated with surfactin (Figure 3C). It was of interest that the treatment by pathogen alone induced the highest increase (2.4 fold) in the *POX* transcripts as early as 24 h and this rapidly declined at 48 h until 72 h). However, no expression of the *POX* gene occurred 24 h after all treatments with CLPs. In similar, the highest *LOX* transcript was also demonstrated in citrus fruit treated with surfactin starting from 48 h and reached its maximum at 72 h (3.68 fold) (Figure 3D). In contrast, fengycin and iturin A treated fruit showed only a small increase of *LOX* transcript at 48 h then this declined 72 h after inoculation. Citrus inoculated with *P. digitatum* alone showed only a slow increment of accumulation from 24 to 72 h (0.4–1.8 fold). It was also of interest, that iturin A showed a small effect on the induction of *LOX* transcript in the citrus flavedo.

### Effects of *B. subtilis* CLPs on activity of defense related enzymes

**Figure 4:**
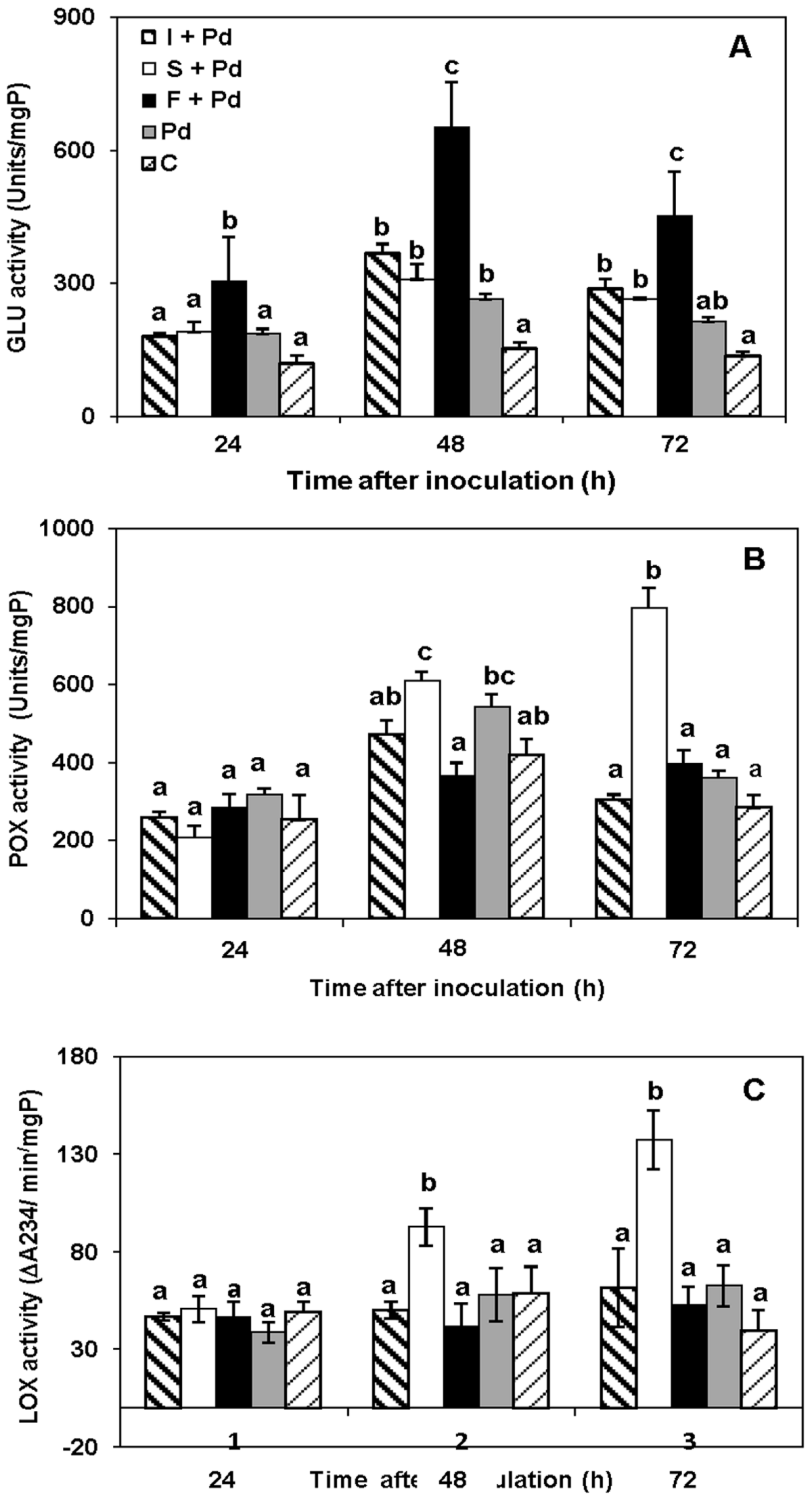
Activity of defense related enzymes induced by various CLPs in flavedo. Activity of A: GLU, B: POX and C: LOX in Valencia flavedo tissue response to various treatments. All treatments of iturin A (I), fengycin (F), and surfactin (S) had been inoculated with *P. digitatum* (Pd) (10^4^spores/mL) and sterile water control (C). In each treatment, vertical bars represent the standard error from the mean value of three replications. Columns with the same letter above them do not differ significantly (*p*≤0.05) by Duncan's multiple range test.

The effects of CLPs on activities of GLU, CHI, POX and LOX were determined in flavedo at 24, 48, and 72 h following inoculation with *P. digitatum*. CHI activity was not detected in the any treated tissues. The pattern of glucanase activity in all treatments was similar, GLU activity was highest during 24–48 h and subsequently declined 72 h after following treatment (Figure 4A). However, GLU activity was significantly higher (*p*≤0.05) in fengycin treated fruit than in fruit treated with surfactin, iturin or *P. digitatum* alone. GLU activity in fruit treated with fengycin was 652.6±37.8 Units/mgP at 48 h, but decreased to 451.2±10.5 Units/mgP at 72 h following treatment. GLU activity in both treatments with surfactin (309.9±33.9 Units/mgP) or iturin A (367.3±21.5 Units/mgP) also increased in citrus flavedo at 48 h and then substantially inclined during 48–72 h after inoculation, but not to the extent that did fengycin.

Patterns of POX and LOX activity in fruit treated with surfactin were similar as seen in Figure 4B and 4C. An increase in LOX and POX activity was also observed with surfactin treatment during at 48 and 72 h following treatment. Activity of POX and LOX was higher in surfactin treatment than other treatments. At 72 h after following treatment, the POX activity in surfactin treated fruit reached a maximum with 796.2±50.4 Units/mgP, and itwas significantly higher than in fengycin, surfactin and *P. digitatum* alone (Figure 4B). Inoculation with *P. digitatum* resulted in an increase in POX activity in surfactin treated citrus flavedo (610±22.0 Units/mgP) at 48 h and remained constant until 72 h (796±50.4 Units/mgP). On the other hand, POX activity in fruit treated with iturin reached a maximum of 470±36.3 Units/mgP at 48 h, but decreased to 303.1±13.8 Units/mgP at 72 h following treatment. As seen in Figure 4C, LOX activity following surfactin treatment increased at 24 h and reached the highest level (137.4±15.2 ΔA234/min/mgP) at 72 h after inoculation. At 48 and 72 h after inoculation, surfactin treatment gave significant difference of LOX activity compared with other CLP treatment. On the one hand, the effects of iturin A, fengycin or *P. digitatum* alone on LOX activity in flavedo were not significant during 24–72 h.

### Synergistic effects of CLPs on abundance of defense related gene transcripts

**Figure 5:**
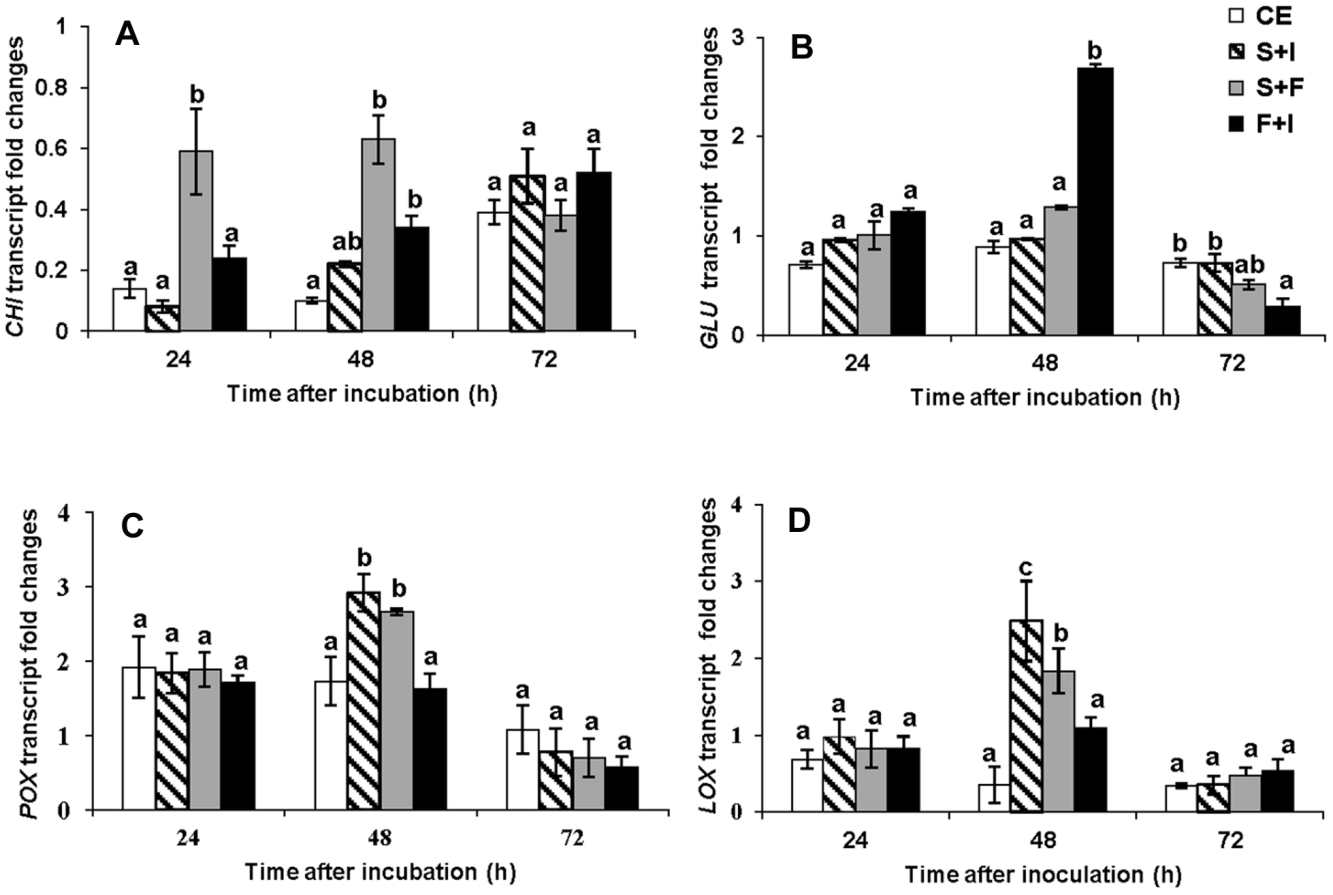
Abundance of transcripts for the defense related genes induced by mixtures of CLPs in flavedo. Expression level of A: *GLU*, B: *CHI*, C: *POX*, and D: *LOX* after wounding and adding the CLPs combination. iturin A (I); fengycin (F); surfactin (S); crude extract (CE). In each treatment, vertical bars represent standard error from mean of three replications. Columns with the same letter above them do not differ significantly (*p*≤0.05) by Duncan's multiple range test.

To determine if there were synergistic effects between iturin A, fengycin, and surfactin on the accumulation of defense related gene transcripts, the abundance transcripts of *CHI*, *GLU*, *POX*, and *LOX* genes were investigated in citrus fruit after treatment with a combination of CLPs without pathogen infection after 24, 48 and 72 h (Figure 5). Low abundance of *CHI* transcripts was presented in all treatments (Figure 5A). However, in citrus fruit treated with fengycin *CHI* transcripts increased and reached a maximum level at 72 h, while *CHI* transcripts in fruit treated with surfactin increased between 24 and 48 h then declined between 48 and 72 h.

The abundance of *GLU* transcripts in flavedo treated with a mixture of fengycin and iturin A showed the highest induction among CLP combinations, reached its maximum (2.8×) at 48 h and markedly decreased between 48 and 72 h (Figure 5B). The abundance of *GLU* transcripts was lower in flavedo that had been treated with either combinations of surfactin and iturin or surfactin and fengycin (0.97× and 1.29×). Crude extract showed the least effect on *GLU* transcript abundance.

At 48 h following treatment (Figure 5C, 5D), the *POX* and *LOX* transcript levels showed a significant increase in flavedo that had been treated with combination of surfactin and iturin A or fengycin. However, a lower induction was seen when iturin A was used in combination with fengycin. Other treatments of citrus fruit showed a similar accumulation pattern at 24 h and they all gradually decreased at 72 h.

### Synergism of CLPs on the induction of defense related enzymes in flavedo

**Figure 6:**
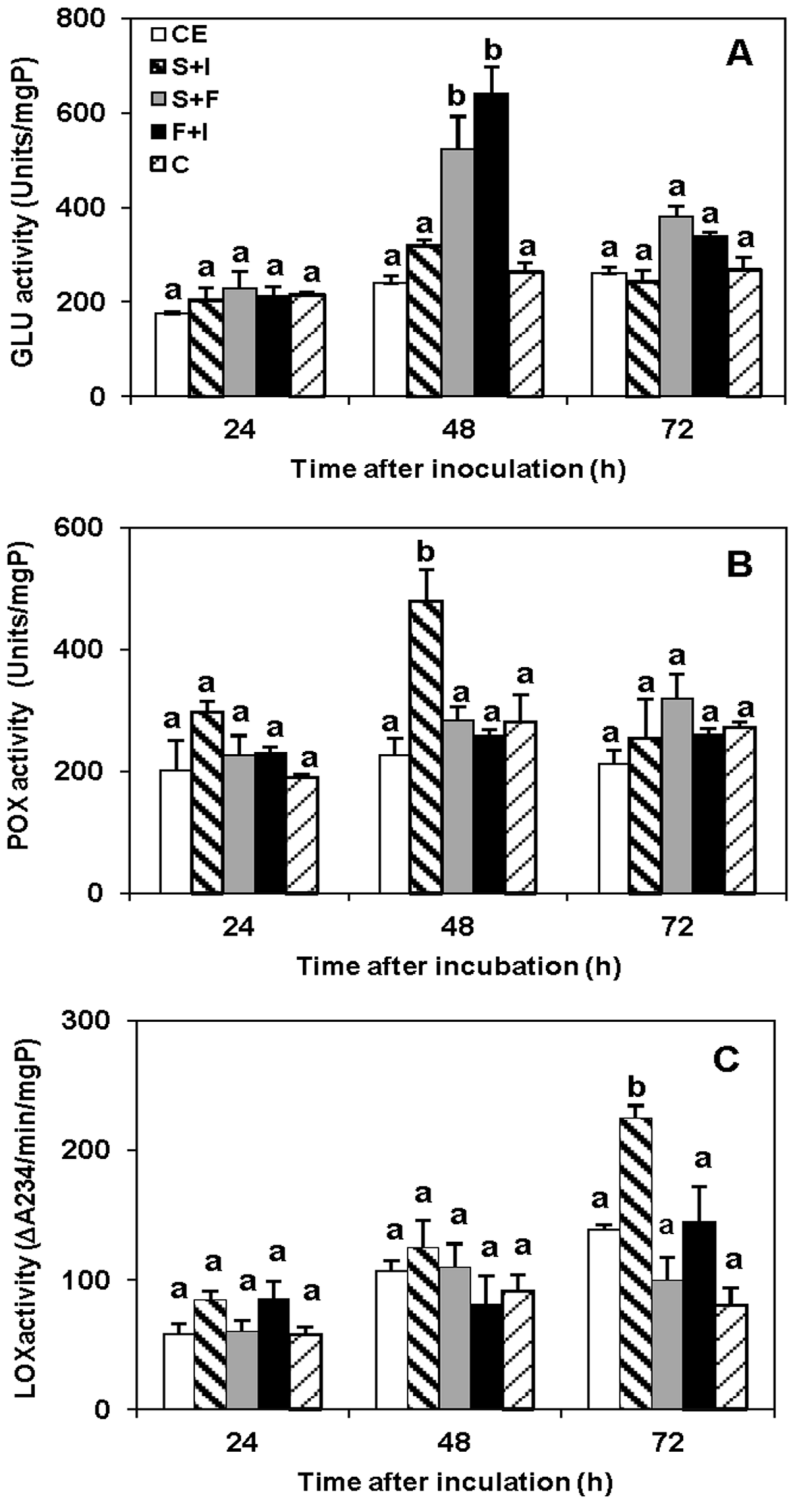
Activities of defense related enzymes induced by mixtures of CLPs in flavedo. Activity of A: GLU, B: POX and C: LOX in Valencia flavedo tissue response to various treatments without pathogen. iturin A (I); fengycin (F); surfactin (S); crude extract (CE); sterile water control (C). In each treatment, vertical bars represent standard error from mean of three replications. Columns with the same letter above them do not differ significantly (*p*≤0.05) by Duncan's multiple range test.

Synergism of CLPs on CHI, GLU, POX, and LOX activities after treatment of the combined CLPs without pathogen infection were presented in Figure 6. CHI activity was not detected in the treated flavedo. GLU activity (Figure 6A) was greatest in flavedo that had been treated with a mixture of fengycin and surfactin or fengycin and iturin A. Glucanase activity was highest at 48 h and then decreased between 48 and 72 h.

POX activity was highest in flavedo that had been treated with a combination of surfactin and iturin A (479±51.3 units/mgP) at 48 h after incubation (Figure 6B). Induction of POX activity was not significantly different in all other treatments. This result indicated that LOX activity increased between 24 and 72 h following CLP treatment (Figure 6C). A combination of surfactin with iturin A produced the greatest increase of LOX activity in citrus flavedo at 72 h. Furthermore, the present data showed that total LOX activity in fruit treated with CLP increased slightly between 24 and 72 h compared to control treatment.

## Discussion

The MALDI-TOF MS analysis has been extensively used for identification of low mass CLPs obtained from bacterial colony and crude extract as shown in previous reports [24]–[25]. The finding of the RP-HPLC profiles of CLPs produced by *B. subtilis* ABS-S14 is consistent with other works [26], and also its CLPs MALDI-TOF MS data is similar to the previous investigations [22],[27]. Hence, the HPLC and MALDI-TOF MS demonstrated in the present study confirms the existence of iturin, fengycin, and surfactin families with multiple isoforms in the CLPs produced by *B. subtilis* ABS-S14.

The ability of antagonist strains of *B. subtilis* to control plant diseases has been attributed to their production of a variety of cyclic lipopeptide antibiotics [28]. Inhibition of green mold decay in citrus fruit after treatment with crude antibiotics produced by antagonistic strains of *B. subtilis* has been demonstrated [13]. To gain more detailed knowledge of the molecular basis of the plant defense mechanisms activated by these microbial antagonists, the abilities of CLPs obtained from *B. subtilis* to elicit defense-related gene expression was determined. Insight of the role of individual CLP produced by *B. subtilis* ABS-S14 on induction of ISR involving enzymes in *P. digitatum* inoculated citrus flavedo, as well as the synergism of CLP itself on the stimulation of citrus tissues responses were subsequently discussed.

Plants respond to biotic stresses by producing several kinds of chemicals and defense proteins that help to protect the plant from pathogen infection. The mechanisms involved in biocontrol activity are based on antibiosis and the triggering of plant immune systems. It is widely known that non-pathogenic antagonistic bacteria and their secondary metabolites can act as biological elicitors to induce a resistance mechanism in plants i.e. the so called ISR response [29]. More recently, the ability of CLPs to elicit expression of defense related genes has been extensively examined and confirmed for a wide variety of plants [30]. It has also been reported that surfactin and fengycin induce defense mechanisms in tomato [3], bean [31], tobacco cells [32], sugar beet [33] and melon [12]. In this study, we have confirmed that fengycin and surfactin produced by the antagonistic *B. subtilis* ABS-S14 elicit the induction and expression of defense-related genes in citrus fruit. Furthermore, fengycin stimulated the genes that encode for GLU, which has a direct effect on the pathogen, whereas surfactin activated the genes coding for POX and LOX, proteins that play important roles in generating signal molecules for activating ISR mechanisms. In contrast, although iturin A was antagonistic towards *P. digitatum*, there was no effect on transcript abundance of the genes investigated in this study. The purified CLPs elicit the expression of defense related genes like *GLU*, *POX*, and *LOX* upon co-application with *P. digitatum*. Clearly, fengycin has a strong effect on the induction of *GLU* gene expression as the transcript levels were much higher than in the treated tissues with the pathogen alone. However, there was only a low induction of the *CHI* gene expression at the transcript level in fruit treated with fengycin was detected in the present study. In similar, recent study mentioned about effect of lipopeptide antibiotics produced by *B. amyloliquifaciens* on chitinase gene activation in sugar beet [33]. In addition, in this work surfactin obtained from *B. subtilis* ABS-S14 was shown to be definitely required for the accumulation of *POX* and *LOX* transcripts in citrus peel. In contrast, different phenomenon have occurred with iturin A which is typically the most potent lipopeptide of the antagonist *B. subtilis* ABS-S14 against *P. digitatum* as reviewed in previous reports [3],[28]. According to a report from Ballester et al. [34], the *P. digitatum* was shown to induce resistance in citrus flavedo one day after inoculation. An increase in activities of PAL, POX, GLU and CHI in conjunction with the increased gene expression was also reported. However, induction of defense-related gene expression in citrus established here was obviously activated by the *B. subtilis* ABS-S14 CLPs at longer time (48 h) after co-application with *P. digitatum*. In parallel, the impact of the enhancement of the corresponding enzyme activity should be essential for the manifestation of a defense mechanism in plants together with the antibiotic role of the CLPs produced by the antagonistic bacteria very early after the invasion by the pathogen. Therefore, delay of citrus fruit decay from a *P. digitatum* infection occurred after treatment with these CLP compounds was seen in the present work.

The mechanisms of action of iturin A, fengycin and surfactin involved in plant protection as elucidated in this work were somewhat similar to those of lipopaenimyxin [35] in that they activated genes that encoded for proteins that responded to the external stimuli: such as cell wall proteins, defense and cell rescue, lipid-related signaling, membrane transport, oxidative stress, phenylpropanoid and phytoalexin pathways, primary metabolism, protein synthesis and their processing in a legume cell suspension. Furthermore, *PR1*, *PR2*, and *PR5* and the jasmonic acid-responsive gene (*vsp*) have been reported to respond to ultrashort lipopeptides in Arabidopsis [36]. The roles of the POX and LOX proteins in plant physiology and the induction of ISR have been previously elucidated. The present findings indicate that *B. subtilis* surfactin activates genes encoding for the LOX and POX proteins and play an important role in generating a signaling molecule for inducing ISR in citrus flavedo in response to *P. digitatum* attack. Previous research has indicated LOX is a key enzyme for JA synthesis [8]. The main process of ISR follows three cascade steps that include: elicitor recognition, systemic signal transduction, and the defense response itself [6]. It also well known that the signaling of the ISR mechanism is controlled by JA and ethylene in all plant species [37] and thereby regulates the expression of all defense related genes. There is strong evidence from previous work to confirm that LOX was related to the generation of a signaling molecule in the ISR pathway and was essential for the stress-induced JA accumulation in plants, acting as an oxylipin, a signalling molecule in the ISR mechanism [38]. In higher plants, POX is associated with disease resistance has a role in regulating the production of antifungal compounds, phenolic metabolic products (lignin), phytoalexins and salicylic acid in response to pathogen infection [39]. Our results demonstrate that POX mRNAs and proteins in flavedo significantly increased when treated with surfactin, while fengycin exhibited a slight effect on POX transcription. It have been reported the phenolics in tuber cells were increased after treatment with fengycin [40]. According to Jourdan et al. [30] early events of the defense related responses, such as the accumulation of phenolics and reactive oxygen species (ROSs) in tobacco cells increased after treatment with surfactins. Several studies have reported that roles for POX in oxidative signal transduction leading to the control of expression of defense-related genes associated with an ISR cascade [41]. It is not surprising that the *P. digitatum* pathogen elicited the expression of *POX* and *LOX* genes as early as 24 and 48 h after inoculation as shown in this study. A possible explanation for this might be that wounding and pathogen infection also activated the expression of the defense-related and the stress gene [42].

Two hydrolytic enzymes; CHI and GLU belong to the PR protein families which are accepted as markers of ISR involved in a general defense response after wounding or attack by a pathogen [11]. This investigation showed a strong gene expression of *GLU* and *CHI* in fruit induced by both fengycin and the pathogen. However, higher levels of *GLU* and *CHI* expressions were obviously shown in fruit treated with the fengycin–pathogen co treatment rather than when treated with the pathogen alone. The present finding seems to be similar to previous investigation that showed that *CHI1* gene expression in citrus was increased after exposing to a lipopeptide [43]. Typically, both enzymes are present at low levels in most plant tissues, but their gene transcriptions are induced by elicitors such as antagonistic agents [44], UV-irradiation, pathogens and JA, and β-aminobutyric acid [45].

## Conclusion

Among the three kinds of CLPs produced by *B. subtilis* ABS-S14, iturin A and fengycin but not surfactin show strong antifungal activity against *P. digitatum*. Surfactin and fengycin trigger plant defenses, but iturin A did not have such a role. These findings clearly confirmed that CLPs obtained from this strain of *B. subtilis* act as effective triggers for disease resistance mechanisms in citrus fruit by initiating defense response through the ISR mechanism in a plant host for protection against the pathogen. However, a detailed analysis of the abilities of CLPs to elicit defense-related genes in citrus flavedo in the absence of infection by pathogens reinforces the biocontrol potential of each of the CLPs produced by this strain of *B. subtilis* that promises to be of use to protect citrus from some fungal infections.
